# Quickly Finding Orthologs as Reciprocal Best Hits with BLAT, LAST, and UBLAST: How Much Do We Miss?

**DOI:** 10.1371/journal.pone.0101850

**Published:** 2014-07-11

**Authors:** Natalie Ward, Gabriel Moreno-Hagelsieb

**Affiliations:** Department of Biology, Wilfrid Laurier University, Waterloo, Ontario, Canada; University of Florida, United States of America

## Abstract

Reciprocal Best Hits (RBH) are a common proxy for orthology in comparative genomics. Essentially, a RBH is found when the proteins encoded by two genes, each in a different genome, find each other as the best scoring match in the other genome. NCBI's BLAST is the software most usually used for the sequence comparisons necessary to finding RBHs. Since sequence comparison can be time consuming, we decided to compare the number and quality of RBHs detected using algorithms that run in a fraction of the time as BLAST. We tested BLAT, LAST and UBLAST. All three programs ran in a hundredth to a 25th of the time required to run BLAST. A reduction in the number of homologs and RBHs found by the faster algorithms compared to BLAST becomes apparent as the genomes compared become more dissimilar, with BLAT, a program optimized for quickly finding very similar sequences, missing both the most homologs and the most RBHs. Though LAST produced the closest number of homologs and RBH to those produced with BLAST, UBLAST was very close, with either program producing between 0.6 and 0.8 of the RBHs as BLAST between dissimilar genomes, while in more similar genomes the differences were barely apparent. UBLAST ran faster than LAST, making it the best option among the programs tested.

## Introduction

The purpose of this work is to evaluate the speed, number and quality of orthologs mapped as reciprocal best hits (RBHs) as detected and scored using NCBI's BLAST [1], [2], the Blast-Like Alignment Tool (BLAT) [3], LAST [4], and UBLAST [5]. The need for this work stems from three main problems in comparative genomics: (i) The exponential increment in the number of genomes available in public databases; (ii) The concomitant need for methods to quickly find homologous sequences in general, and orthologs in particular, across available genomes (for definitions see below); (iii) The appearance of faster software for sequence comparison whose adequacy for particular tasks compared to commonly used software should be assessed.

Several research groups have made orthologs available through web services to a wider community (see for example: [6]–[12]). However, particular researchers might still prefer to make their own calculations due to reasons such as those that we have listed before [13]: (a) researchers' own newly sequenced genomes under analyses; (b) a need for updated ortholog mappings not available in published ortholog databases; (c) lack of agreement about the genome annotations to use, for instance, those provided by the authors of a genome, corrections such as those within the RefSeq database [14], [15], the HAMAP project [16], [17], or even those re-annotations produced by other research groups (*e.g.* [18]–[21]).

Orthologs, which could be referred to as the “same genes” in different species, are defined as homologous genes diverging after a speciation event [22]. Because of this evolutionary relationship, orthologous genes are expected to keep their original functions. Paralogs, defined as homologous genes diverging after a duplication event [22], have been proposed as a source of functional innovation [23], [24], and are therefore less expected to have similar functions. Since it seems safer to infer similar functions between orthologs than between paralogs [25]–[28], it is important to be able to differentiate between orthologs and extra-paralogs, paralogous genes residing in different organisms [29].

Evidently, the definitions provided above are based on the event separating the histories of the genes in question. In practice, researchers rely on sequence similarity and suitable statistics for detecting homologs. After detecting putative homologs, producing evolutionary models such as phylogenetic trees, though performed by some groups (*e.g.* [30]–[32]), would be too computationally intensive to run in order to differentiate between orthologs and paralogs across available genomes. The growth of the sequence databases does not make a phylogenetic approach practical. Thus, most research in comparative genomics relies on shortcuts, or working definitions, for orthology. Probably the most common working definition of orthology is that of Reciprocal Best Hits (RBH) [33], [34], whereby two genes residing in two different genomes are deemed orthologs if their protein products find each other as the best hit in the opposite genome.

The task of finding homologs to a sequence of interest (the *query*) in a database containing many other sequences (the *subjects*) can be conceptualized as getting the best possible alignment of the query against all the subjects, scoring each of these alignments, and choosing those whose scores surpass a given threshold, or that comply with some alignment statistic. An exhaustive process using the dynamic programming algorithm by Smith and Waterman [35] could be so time consuming that researchers have developed heuristic algorithms. One of these heuristic algorithms, BLAST [36], has been the program of choice to compare proteins and therefore to produce RBHs, because of its speed compared to the exhaustive algorithm mentioned above, and to another heuristic algorithm, namely FASTA [37].

However, the constant increase in genomic sequences make it increasingly harder to rely on BLAST. With the pressure for faster results, other authors have produced faster heuristic algorithms. Among them, the most commonly used ones seem to be the BLAST-Like alignment Tool (BLAT) [3] and UBLAST [5], with the most recent addition of LAST [4]. These programs implement an indexed subject database, which allows to quickly find the most promising proteins to align; they use different methods to seed a pairwise alignment, such as stretches of identical amino-acid residues in BLAT, or variable size seeds implemented into a suffix tree in LAST; and they quickly drop the search for further protein comparisons to avoid wasting time on less likely matches. Further details can be found in the respective references and manuals [3]–[5]. While these programs run in a fraction of the time required to run BLAST, the speed comes at the cost of missing some matches otherwise found by BLAST.

In this work we used the genomes of four organisms: *Escherichia coli* K12 [38], *Bacillus subtilis* [39], *Methanosarcina mazei* Go1 [40], and *Saccharomyces cerevisiae* [41], as query genomes and a database of around 2750 genomes, to compare the speed, the number and the quality of orthologs found as RBH using four programs to finding similar protein sequences; namely, NCBI's BLAST, LAST, UBLAST, and BLAT.

## Results and Discussion

### The number of RBHs found decreases from BLAST to LAST to UBLAST to BLAT

**Figure 1:**
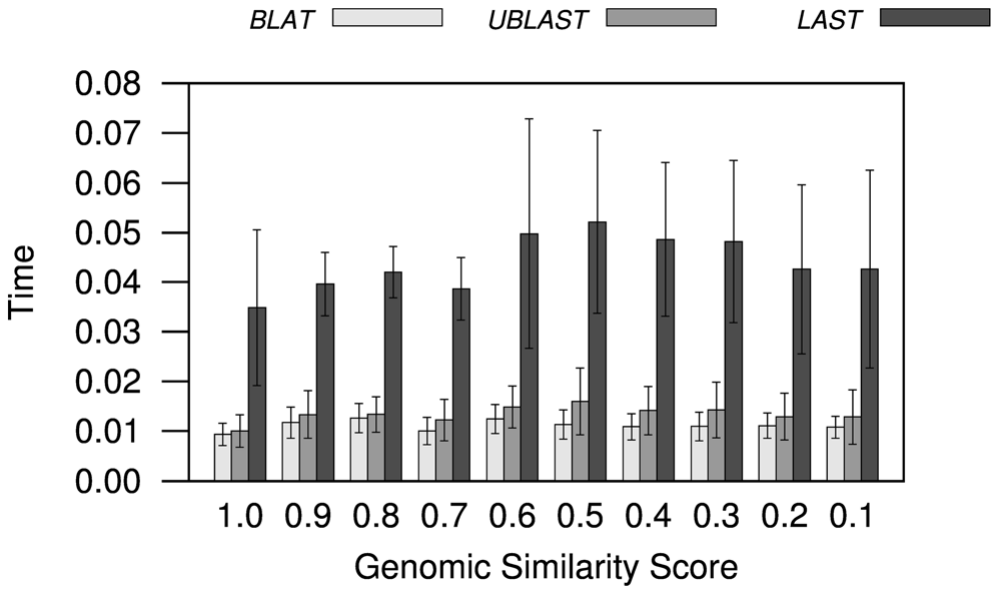
Difference in run time. Both UBLAST and BLAT ran in about a hundredth of the time as NCBI's BLAST, while LAST ran in about a 25th of the time required for BLAST. Note: as in Figures 2 and 4, the bars represent averages for pairwise genome comparisons involving genomes binned at intervals of 0.1 of Genomic Similarity Score (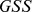); the number of genomes at each 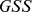 bin is not the same; and the error bars show standard deviations representing the variability of results among the genomes at each bin.

Both BLAT and UBLAST ran in close to a hundredth of the time taken by NCBI's BLAST, while LAST ran in about a 25th of the time required for BLAST (Fig. 1). LAST was the program showing the most variation in time to run when compared to BLAST, as well as the most variation in numbers of homologs and reciprocal best hits found. This is probably due to LAST's use of adaptive alignment seeds, similar ‘words’ shared by sequences, in its strategy for quickly finding sequences that might produce a significant alignment. Adaptive seeds will be different in length and effectiveness across different databases. Thus, LAST's results with different sequence databases should vary the most when compared to results with BLAST, than results obtained using tools whose difference to the way BLAST works is more constant. For example, BLAT normally searches for identical ‘tiles’ of length 5 when comparing proteins before attempting an alignment.

**Figure 2:**
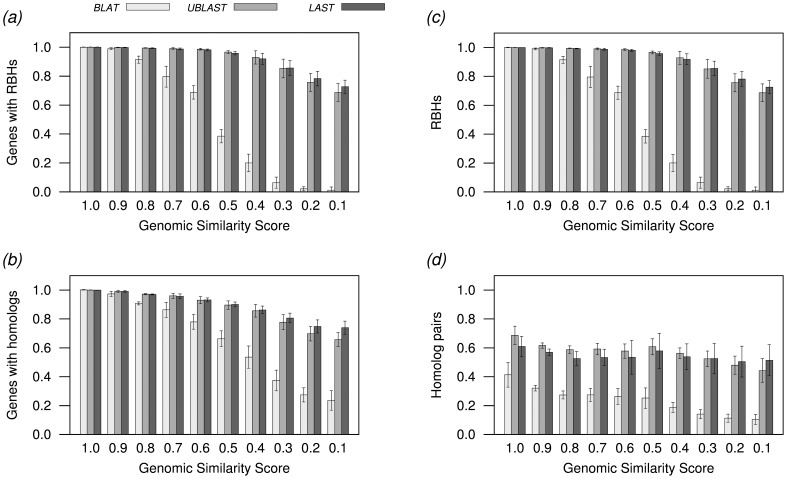
Differences in number of homologs and reciprocal best hits (RBHs). All numbers were normalized against the corresponding numbers obtained with NCBI's BLAST. As expected, the faster programs found fewer RBHs than BLAST. Such differences become more evident as the similarity between genomes decreases (as the genomic similarity score, 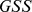, decreases). This effect was much more pronounced for BLAT. The number of genes finding RBHs (a) and the total RBHs (c) was so small that the two graphs are almost identical. However, the differences between genes finding homologs and the total number of homologous pairs is much more apparent. The number of homolog pairs was always smaller for the fastest programs than for BLAST, suggesting that a good proportion of the differences in RBHs found is due to a lower search depth by the faster programs. See Note in Figure 1.

The programs tested can be ordered from the one producing the highest number of RBHs to the one producing the lowest number of RBHs as BLAST>LAST>UBLAST>BLAT (paired t-tests 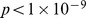; Table set S1 and Table set S2). As it might be expected, the decrease in the number of RBHs found by the faster programs (LAST, UBLAST and BLAT) becomes more pronounced with the overall dissimilarity between the genomes compared (Fig. 2a, 2b; Table sets S1 and S2). The lowest proportion of RBHs found by LAST was close to 0.8 of those found by BLAST, while for UBLAST it was between 0.6 and 0.7. However, these proportions remained very close to 1 in other, more similar, genomes. Given that BLAT is optimized for quickly finding very similar nucleotide sequences [3], it was the program producing the lowest number of RBHs. BLAT showed a quick loss of sensitivity with genome dissimilarity. The program did not find RBHs for a few genome comparisons, and found just a few RBHs between distantly related genomes (Tables with RBHs and homologs are available at: http://microbiome.wlu.ca/Orthologs/).

We also accounted for the number of genes finding homologs (Fig. 2b; Table set S1) and the number of homologous pairs (a gene can have more than one match, and therefore could produce more than one homologous pair) (Fig. 2d; Table set S2). The number of genes finding homologs showed similar tendencies as the number of RBHs above suggesting that the differences in the number of RBHs found is related to a corresponding difference in the number of genes finding homologs. UBLAST had a tendency to find a higher proportion of genes with RBHs per gene finding a homolog than any other program (paired t-tests 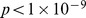), while BLAT had a tendency to find the fewest RBHs per gene finding a homolog (Fig. 2a, 2b). The number of homologous pairs was always smaller for the fastest programs (Fig. 2d). UBLAST and LAST produced the highest proportion of RBHs per homologous pair, while BLAT produced the lowest proportion of RBHs per homologous pair (Fig. 2c, 2d). These results suggest that another source or differences in RBHs is the search depth. UBLAST and LAST would have smaller sources of conflict to decide RBHs than BLAST. However, if the number of homologous pairs is too low, as it is in BLAT, then the reciprocal results might be lacking and RBHs might not be found.

### Homologous pairs found by BLAT, UBLAST and LAST are subsets of those found by BLAST

**Figure 3:**
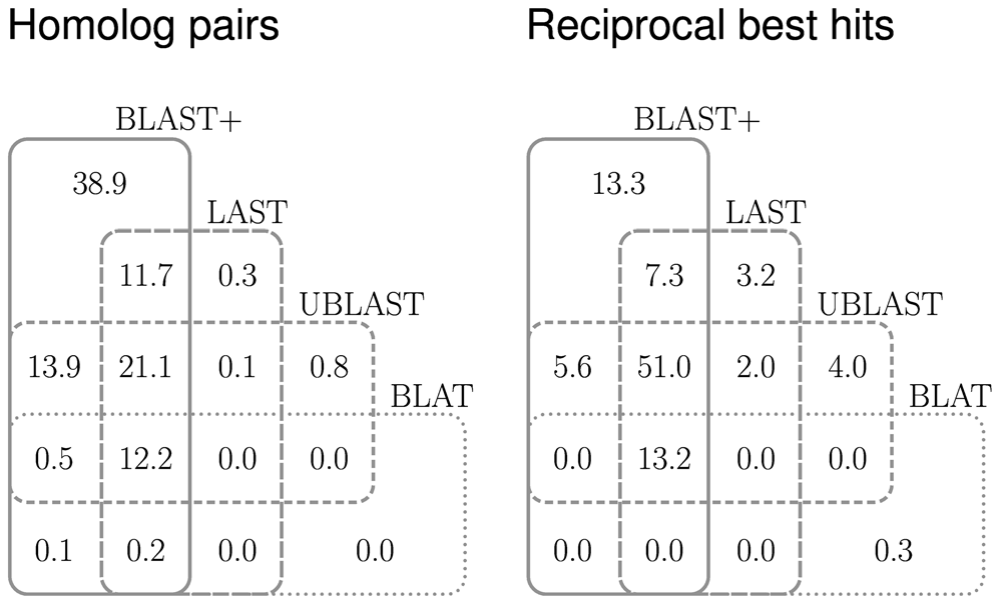
Matching pairs. Most homologous pairs found by LAST, UBLAST and BLAT were also found by BLAST. Differences in scoring resulted in a higher proportion of different RBHs found by either programs than would be expected from the sets of homologous pairs.

As expected, BLAT, UBLAST and LAST found fewer homologous pairs than BLAST did (Fig. 2b, 3), and most of the matching pairs found by the faster algorithms were subsets of those found by the slowest (Fig. 3), with only 1.2% of the total homologous pairs not being detected by BLAST (0.3% found only by LAST, plus 0.8% found only by UBLAST, and 0.1% found by both UBLAST and LAST—note the intersection between the results of these two programs in the Venn diagram).

The Venn diagram on RBHs, however, was not what would be expected from that of the homologous pairs (Fig. 3). Of the total RBHs found by all programs combined, 3.2% were detected only by LAST; 4% were detected only by UBLAST, and 2% were detected by both UBLAST and LAST, but not by either of BLAST and BLAT. Of the same total RBHs found by all programs, 0.3% were detected only by BLAT. The difference in comparison to the homologous pairs results is most probably due to differences in the scoring systems, since, for example, BLAST modifies its scores by taking into account edge effects, and compositional biases in the sequences being compared [42]–[45].

### The error rates are highest with BLAT

To estimate error rates, we analyzed conservation of adjacency of homologous genes (synteny). Conservation of gene order has been previously suggested to be of limited use for the assignment of orthology in prokaryotes due to the high divergence of gene order prevailing in these organisms [34]. However, synteny can still be used to test for the relative quality of predicted orthologs [13], [46], [47].

**Figure 4:**
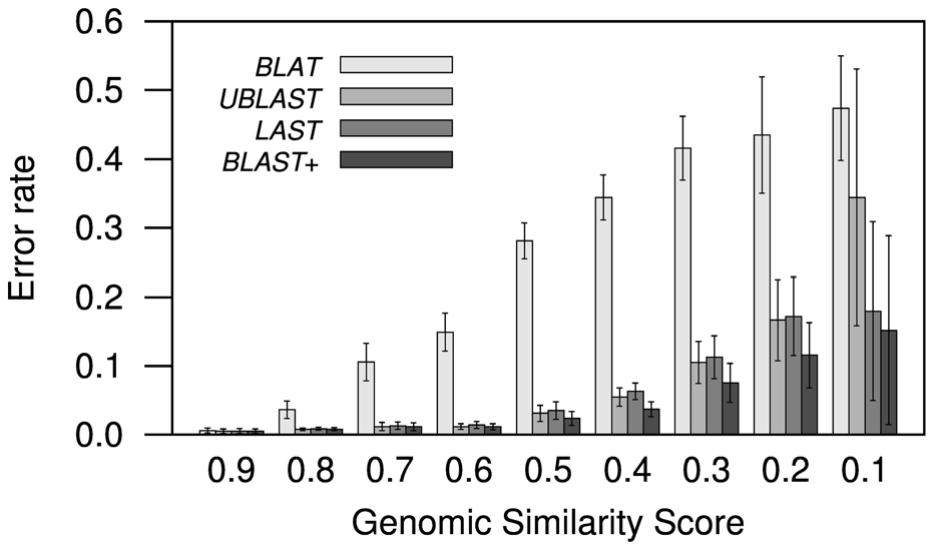
Error rate estimated using conservation of gene order. The estimate consists on false negatives (a paralog conserved next to a RBH) divided by the sum of false negatives + true positives (RBHs showing conservation of gene order). BLAST consistently showed the lowest error rates. Both LAST and UBLAST showed the most similar error rates to those produced by BLAST except when the genomes compared had low 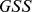, where UBLAST had higher error rates than LAST. BLAT showed the highest error rates. See Note in Figure 1.

The error rates increased with the evolutionary distance as measured using Genomic Similarity Scores (Fig. 4; Table set S3). These error rates were more similar for RBH produced between closely related genomes (Fig. 4). Error rates using LAST and UBLAST were similar to those produced by BLAST, except between the least similar genomes (Fig. 4; Table set S3), where both programs showed higher error rates than BLAST, and UBLAST having higher error rates than LAST among the most divergent genomes. BLAT consistently produced the highest error rates. Though error rates across the board are high among more divergent genomes, we must bear in mind that errors might relate to biologically meaningful events whose probability increases with divergence. For example, gene conversions (recombination between homologous genes), or gene divergences so high that their status as orthologs or extra-paralogs are barely discernible. One more biological source of confusion might be the possibility of co-orthology, which occurs when a duplication event happens after a speciation event [22]. It is possible that some apparent errors arise from divergent co-orthologs that therefore produce slightly different results. However, since the background biological events should be the very same, the difference in error rates should still reflect differences in the results obtained with each program.

### Concluding remarks

We tested three programs that run considerably faster than BLAST for the task of detecting reciprocal best hits (RBHs). These programs have options that alter their default running methods in ways that might improve their performance in terms of sensitivity and thus increase the proportion of homologs found when compared to BLAST. Such increase might result in a concomitant increase in detection of RBHs. However, changing those options also increases the time required to run these programs. Thus, playing with options to try and attain results somewhat more similar to those obtained with BLAST would defy the purpose of this work.

While evaluating the results presented, we must bear in mind that none of the programs tested, not even NCBI's BLAST, was designed for the task of finding reciprocal best hits. The results show that, as would be expected from programs that miss homologs otherwise found by BLAST, the number of RBHs found by LAST, UBLAST and BLAT are mostly a subset of those found by NCBI's BLAST. When dealing with the most dissimilar genomes, both LAST and UBLAST kept between 0.6 and 0.8 of the number of RBHs found by BLAST. Results with more closely related genomes were more similar to those produced using BLAST. Given that BLAT is optimized for finding very similar sequences quickly, it should not be surprising that it missed most of the RBHs between the least similar genomes, and a high proportion of RBHs in the rest. Overall, UBLAST might be the best compromise between speed and sensitivity of the faster programs tested.

## Methods

In this work we used the genomes of *Escherichia coli* K-12 MG1655 (uid57779) [38], *Bacillus subtilis* 168 (uid57675) [39], *Methanosarcina mazei* Go1 (uid57893) [40], and of *Saccharomyces cerevisiae* (uid128) [41], as testing genomes, and compared their annotated protein sequences against those annotated in the 2754 prokaryotic and fungal genomes available at the RefSeq database [14], [15] (ftp://ftp.ncbi.nih.gov/genomes/Bacteria/) by the end of December, 2013. We calculated Genomic Similarity Scores (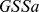) as described previously [29], [48], [49]. Briefly, the 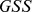 is the normalized BLAST bit score of all reciprocal best hits between any two genomes. In this work *GSS*s were calculated with RBHs produced from NCBI's BLAST results.

The protein sequence comparisons were performed using four programs: (i) NCBI's BLAST version 2.2.28+ [2], which is the BLAST suite of programs implemented in C++; (ii) LAST version 393 [4]; (iii) UBLAST, as implemented in the sequence analysis multitool USEARCH (version 7.0.1001) [5]; and (iv) the BLAST-Like Alignment Tool (BLAT) [3] version 35. We compiled all these programs at 64 bits, except for USEARCH, which is kindly provided by the author precompiled at 32 bits for academic use. All sequence comparisons were run with testing genomes as queries and database genomes as subjects, as well as database genomes as queries and testing genomes as subjects (reciprocal sequence comparisons).

The specific command lines used to run each program are presented in Table 1. The options for NCBI's BLAST different to the defaults were a maximum *E-value* threshold of 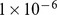 (*-evalue 1e-6*), and a final Smith-Waterman alignment (*-use_sw_tback*). For UBLAST we also specified an e-value threshold of 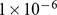 (*-evalue 1e-6*). Since LAST and BLAT do not offer an option to control e-value thresholds, they were run with default values only (BLAT's minimal score is 30, and minimal identity is 25%). However, BLAT calculates an e-value when the output sequence is specified as "blast8" (*-out = blast8*). LAST's e-values can be estimated using the command *lastex* from this program suite. We therefore filtered BLAT and LAST results using their calculated e-values during the process of finding reciprocal best hits. We also required coverage of at least 50% of any of the protein sequences in the alignments.

**Table 1:** Command lines used to run each genome to genome comparison.

Command lines
blastp -num_threads 2 -evalue 1e-6 -use_sw_tback -outfmt 6 -query query_genome -db subject_genome > result
lastal -f 0 subject_genome query_genome.faa > result
usearch7 -ublast query_genome.faa -db subject_genome.udb -evalue 1e-6 -blast6out result
blat -prot subject_genome.faa query_genome.faa -out = blast8 result

Finding best hits involved sorting the results for a query-genome-to-subject-genome comparison from highest to lowest score. The first hit for each query protein within the sorted results would therefore be the best hit. If the next hit had the very same score there would be more than one best hit (the method can therefore produce co-orthologs). We performed the very same procedure for the results ran in the opposite direction. That is, for the results where the subject genome was used as a query, and the query genome was used as a subject. Finally, to find orthologs as reciprocal best hits, for each best hit found by a query protein in the first direction, we checked if it found this query gene as a best hit in the opposite direction.

To estimate error rates in orthology detection, we used a test based on synteny [13], [46], [47]. For every pair of adjacent genes in the testing genomes, we found pairs of corresponding adjacently conserved homologs in any other genome. We then checked if those conserved homologs were also RBHs. If both conserved homologs were RBHs, the pair was considered to consist of two true positives (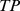). If one gene was a RBH, but the other was not, then we counted the former as a 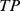, and the latter as a false negative (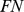). With these definitions, error rates (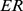) were calculated as:
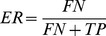



Note that despite this measure might not test for other measures of quality, like true negatives and false positives, the aim here is not to produce a standard error rate, but to compare the relative quality of results among the programs tested.

## Supporting Information

Table Set S1 These tables contain counts for genes finding reciprocal best hits and genes finding homologs organized on a per query genome fashion. The directory contains the R-script used to run the t-tests comparing the results from each program, and a table with the results of these t-tests.
(ZIP)

Table Set S2 These tables contain reciprocal best hit counts and homolog pair counts organized on a per query genome fashion. The directory contains the R-script used to run the t-tests comparing the results from each program, and a table with the results of these t-tests.
(ZIP)

Table Set S3 These tables contain counts for error estimates organized on a per query genome fashion. The directory contains the R-script used to run the t-tests comparing the results from each program, and a table with the results of these t-tests.
(ZIP)
